# Rational design of binder-free noble metal/metal oxide arrays with nanocauliflower structure for wide linear range nonenzymatic glucose detection

**DOI:** 10.1038/srep10617

**Published:** 2015-06-12

**Authors:** Zhenzhen Li, Yanmei Xin, Zhonghai Zhang, Hongjun Wu, Peng Wang

**Affiliations:** 1 School of Chemistry and Molecular Engineering, East China Normal University, 500 Dongchuan Road, Shanghai 200241, China; 2 Provincial Key Laboratory of Oil & Gas Chemical Technology, College of Chemistry & Chemical Engineering, Northeast Petroleum University, Daqing 163318, China; 3 Water Desalination and Reuse Center, Biological and Environmental Sciences and Engineering Division, King Abdullah University of Science and Technology, Thuwal, Saudi Arabia

## Abstract

One-dimensional nanocomposites of metal-oxide and noble metal were expected to present superior performance for nonenzymatic glucose detection due to its good conductivity and high catalytic activity inherited from noble metal and metal oxide respectively. As a proof of concept, we synthesized gold and copper oxide (Au/CuO) composite with unique one-dimensional nanocauliflowers structure. Due to the nature of the synthesis method, no any foreign binder was needed in keeping either Au or CuO in place. To the best of our knowledge, this is the first attempt in combining metal oxide and noble metal in a binder-free style for fabricating nonenzymatic glucose sensor. The Au/CuO nanocauliflowers with large electrochemical active surface and high electrolyte contact area would promise a wide linear range and high sensitive detection of glucose with good stability and reproducibility due to its good electrical conductivity of Au and high electrocatalytic activity of CuO.

Accurate detection of glucose level in blood is essential for clinical diagnostics in diabetes control^1^,^2^,^3^,^4^. Traditionally, glucose concentration is monitored by an amperometric system, in which glucose is enzymatically oxidized by highly selective glucose oxidase (GOx) immobilized on electrode surface and the thus-generated electrons or reaction product, hydrogen peroxide (H_2_O_2_), is subsequently measured so to determine the concentration of the glucose^5^,^6^,^7^,^8^. By the nature of enzyme, the GOx-based glucose sensor possesses selectivity, but its application is still limited due to the inherent drawbacks associated with enzyme purification, immobilization and its protection from denaturing. Moreover, the great distance between the deeply embedded flavin group (FAD) redox center of GOx and the electrode surface complicates the system and necessitates certain electron shuttles be present in the samples, which is arguably the biggest barrier that limits the sensitivity of this method^9^,^10^. To this end, nonenzymatic, direct electrocatalytic detection of glucose has recently garnered significant interest as it promises an electron-transfer-shuttle-free sensor and thus a high sensitivity and repeatability^11^,^12^.

For a successful nonenzymatic electrocatalytic detection of glucose, high conductivity and catalytic activity is required for the electrocatalyst. Among all of the candidate materials for the electrocatalyst in glucose detection, including noble metal^13^,^14^,^15^, metal oxide^16^,^17^,^18^, carbon materials^19^,^20^,^21^, mesoporous alloy^22^, and polymers^23^,^24^, noble metal and metal oxide materials have distinguished themselves. Recently, various nanostructures, such as nanorod^25^, nanowire^26^, nanotube^27^,^28^, dendritic^29^ and mesoporous materials^30^,^31^, have received significant attention due to their high surface area, efficient charge separation, etc., which are beneficial to many applications. Among the synthesis methods for fabricating one-dimensional nanostructures, anodization stands out owing to its versatility, one step, low-cost, and more importantly seamless connection between the metal substrate and the anodized nanostructures which literally makes the so-prepared nanostructures an ideal electrode with high conductivity^32^,^33^,^34^,^35^. Generally, in a conventional electrochemical nonenzymatic sensing process, the electrocatalysts are prepared in the form of nanoparticles, and are then immobilized on conductive substrates with the help from certain polymeric binders, which are usually insulating and electrochemically inactive. The presence of the polymeric binders in the conventional systems inevitably increases the series resistance, block the otherwise catalytically active sites, and impede the electrolyte diffusion, ultimately leading to a significantly reduced electrocatalytic activity and poor performance of the sensors. For the above-mentioned reasons, anodized one-dimensional nanostructures can be a rational solution to the problem in the conventional systems.

In this study, we propose to utilize anodized one-dimensional nanostructure of metal-oxide and noble metal composite for nonenzymatic glucose detection. The composite of metal-oxide and noble metal is a judicious choice as it would present a good electrocatalytic performance plus conductivity and high catalytic activity inherited from noble metal and metal oxide respectively. As a proof of concept, we synthesized gold and copper oxide (Au/CuO) composite with one-dimensional nanostructure. Among the common noble metals, Au presented highly stable, conductive properties, and good catalytically activity^36^,^37^, in addition, the detailed catalytic mechanism of glucose on gold substrate and operable strategy for improving selectivity and anti-interference ability have well been proposed and determined by Pasta and Cui^38^,^39^, which distinguished itself as best choice for constructing a nonenzymatic sensor. For metal oxides, the CuO was selected due to its high catalytic activity, low cost, abundance of copper in the crust of the Earth^40^,^41^, and more importantly the fact that one-dimensional CuO nanowire structure could be facilely synthesized by anodization^42^,^43^. The Au nanoparticles were decorated on the anodized CuO nanowire array with a simple photo-reduction method, taking on a unique nanocauliflowers structure. Due to the nature of the synthesis method, no any foreign binder was needed in keeping either Au or CuO in place. To the best of our knowledge, this is the first attempt in combining metal oxide and noble metal in a binder-free style for fabricating nonenzymatic glucose sensor. The Au/CuO nanocauliflowers with large electrochemical active surface and high electrolyte contact area would promise a wide linear range and high sensitive detection of glucose with good stability and reproducibility due to its good electrical conductivity of Au and high electrocatalytic activity of CuO.

## Results

### Preparation of Au/CuO nanocauliflower composite

**Figure 1:**
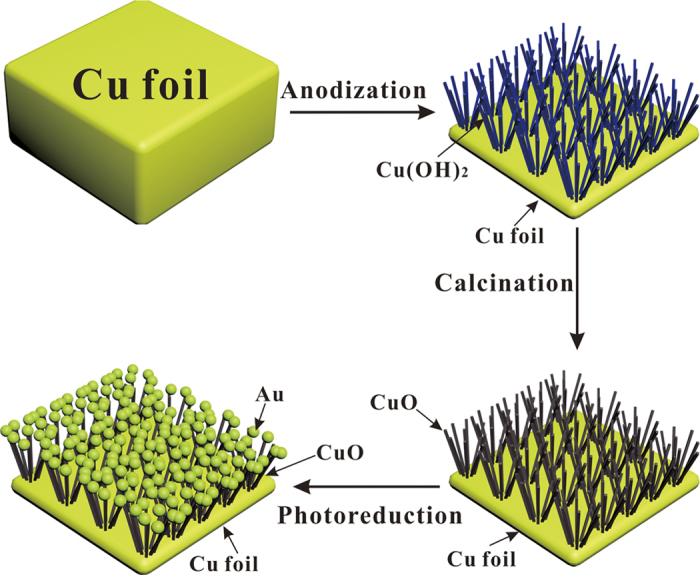
A schematic illustration of preparation of Au/CuO nanocauliflower composite.

The preparation procedures for Au/CuO nanocauliflower is illustrated in Fig. 1. First, he cleaned copper foil was electrochemically anodized in a conventional three-electrode system to form Cu(OH)_2_ nanowire arrays, and then annealed at 180 °C for 1 h to convert Cu(OH)_2_ to CuO with the nanowire arrays well preserved. Second, the Au nanoparticles were deposited onto the CuO nanowires by a direct photocatalytic reduction of HAuCl_4_ in aqueous solution, forming a unique nanocauliflower structures. For comparison, Cu(OH)_2_, CuO, and Au/Cu(OH)_2_ composite electrode were also prepared following otherwise the same procedure.

### Materials Characterization

**Figure 2:**
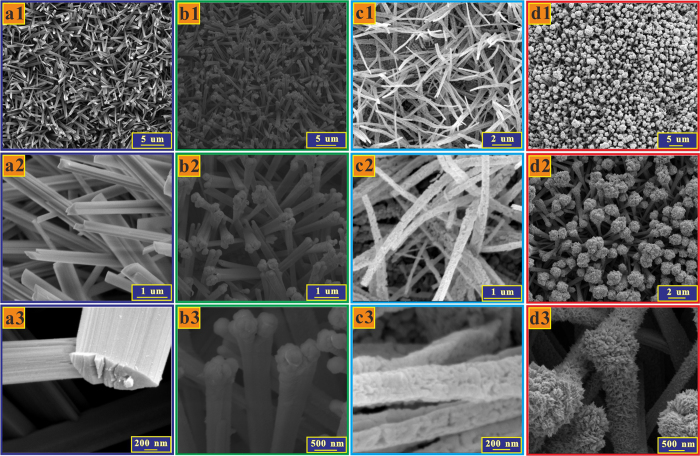
SEM image. (**a**) Cu(OH)_2_ nanowires, (**b**) Au/Cu(OH)_2_ nanowires, (**c**) CuO nanowires, and (**d**) Au/CuO nanocauliflowers in various magnifications.

Figure 2a-d present the scanning electron microscopy (SEM) images of the Cu(OH)_2_ nanowires, Au/Cu(OH)_2_ nanowires, CuO nanowires, and Au/CuO nanocauliflowers respectively. As can be seen, a dense layer of the Cu(OH)_2_ nanowires covered the Cu foil substrate after anodization (Fig. 2a1-a3) and with the deposited Au nanoparticles on top, the Cu(OH)_2_ was able to maintain the uniform nanowire structures (Fig. 2b1-b3). After annealing, the bluish Cu(OH)_2_ nanowires turned into black CuO nanowires with somewhat rough surfaces (Fig. 2c1-c3). The length of the CuO nanowires was around 8~10 μm (Supplementary Fig. S1). The energy dispersive X-ray spectroscopy (EDS) spectra of the Au/CuO present evidence of the successful Au loading (Supplementary, Fig. S2). As shown in Fig. 2d1-d3, different from the Au/Cu(OH)_2_ case, for the Au/CuO, the Au fully covered the entire CuO nanowire surfaces, which may be ascribed to the rough CuO nanowire surfaces being active Au loading sites. More interestingly, there were Au nano-aggregates on top of each nanowire tips due to spatial availability, which presented a uniform nanocauliflowers structure (Supplementary, Fig. S3). It is worth mentioning that the pH of the aqueous HAuCl_4_ solution had a significant effect on the Au deposition and in this work, an optimized pH value of 6.5 was used in both Au/Cu(OH)_2_ and Au/CuO cases. It was found that a lower pH value would result in the removal of the nanowires from the copper foil substrate (Supplementary Fig. S4) while a higher pH value would fail to deposit Au on the nanowires (Supplementary Fig. S5).

**Figure 3:**
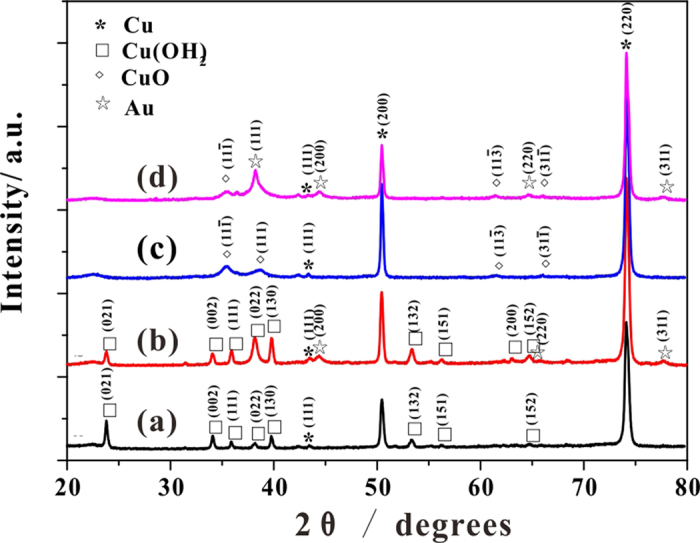
XRD patterns. (**a**) Cu(OH)_2_, (**b**) Au/Cu(OH)_2_, (**c**) CuO and (**d**) Au/CuO.

The crystal structures of the Cu(OH)_2_, Au/Cu(OH)_2_, CuO, and Au/CuO samples were characterized and successfully confirmed by X-ray diffraction (XRD). As shown in Fig. 3, there are a weak diffraction peak at 43.3° and two strong diffraction peaks at 50.4° and 74.1° which come from the copper foil substrate (JCPDS 04-0836). For the Cu(OH)_2_ sample, the diffraction peaks indexed to orthorhombic Cu(OH)_2_ (JCPDS 80-0656) could be clearly identified. While the diffraction peaks at 35.5°, 38.7°, 61.5° and 66.2° in the CuO sample can be assigned to the (11-1), (111), (11-3), and (31-1) planes of CuO phase (JCPDS 48-1548), with no sharp peak that can be indexed to other impurities indicating high purity of the CuO. Particularly, both of the Au/Cu(OH)_2_ and Au/CuO samples present diffraction peaks at 38.2°, 44.3°, 64.5°, and 77.5°, which can be assigned to the (111), (200), (220), and (311) planes of Au phase (JCPDS 65-2870), with the (111) plane of Au significantly overlapping with the (022) plane of Cu(OH)_2_ and (111) plane of CuO. The average crystalline sizes of Au were estimated from the Debye-Scherrer equation from the (200) crystal plane with 13.8 nm and 12.4 nm for Au/Cu(OH)_2_ and Au/CuO respectively^44^.

**Figure 4:**
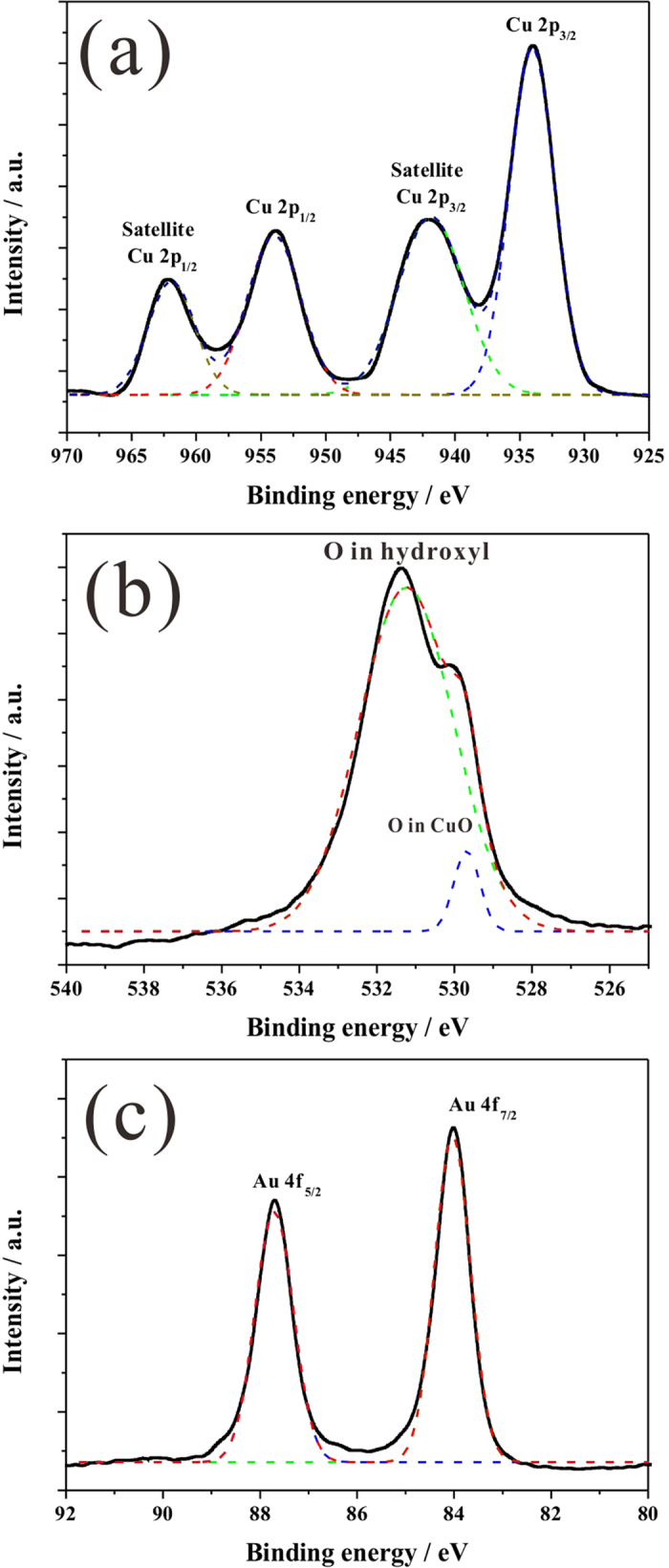
High resolution XPS of Au/CuO sample. (**a**) Cu 2p, (**b**) O 1s and (**c**) Au 4f.

The X-ray photoelectron spectroscopy (XPS) survey spectra of the Au/CuO clearly indicate Cu, O and Au elements, with no other impurities being determined (Supplementary Fig. S6a, b). The XPS of Cu 2p core level is presented in Fig. 4a where two peaks located at 934.0 and 954.0 eV are shown, which can be assigned to the binding energy of Cu 2p_3/2_ and Cu 2p_1/2_ respectively, indicating the presence of the Cu^2+^ on the sample^45^. In addition, two extra shake-up satellite peaks for Cu 2p_3/2_ and Cu 2p_1/2_ at 941.8 and 961.9 eV are also observed on higher binding energy side, implying the presence of an unfilled Cu 3d^9^ shell and thus further confirming the existence of Cu^2+^ on the sample surface. The core-level XPS of O 1s in Fig. 4b presents two band peaks at 529.7 and 531.2 eV, which can be ascribed to oxygen in CuO lattice and hydroxyl adsorption on its surface^46^. The Au 4f core level from the Au/CuO (Fig. 4c) can be fitted with two peaks at a binding energy of 84.0 and 87.7 eV, corresponding to Au 4f_7/2_ and Au 4f_5/2_ respectively, which is attributed to metallic gold Au(0)^47^.

**Figure 5:**
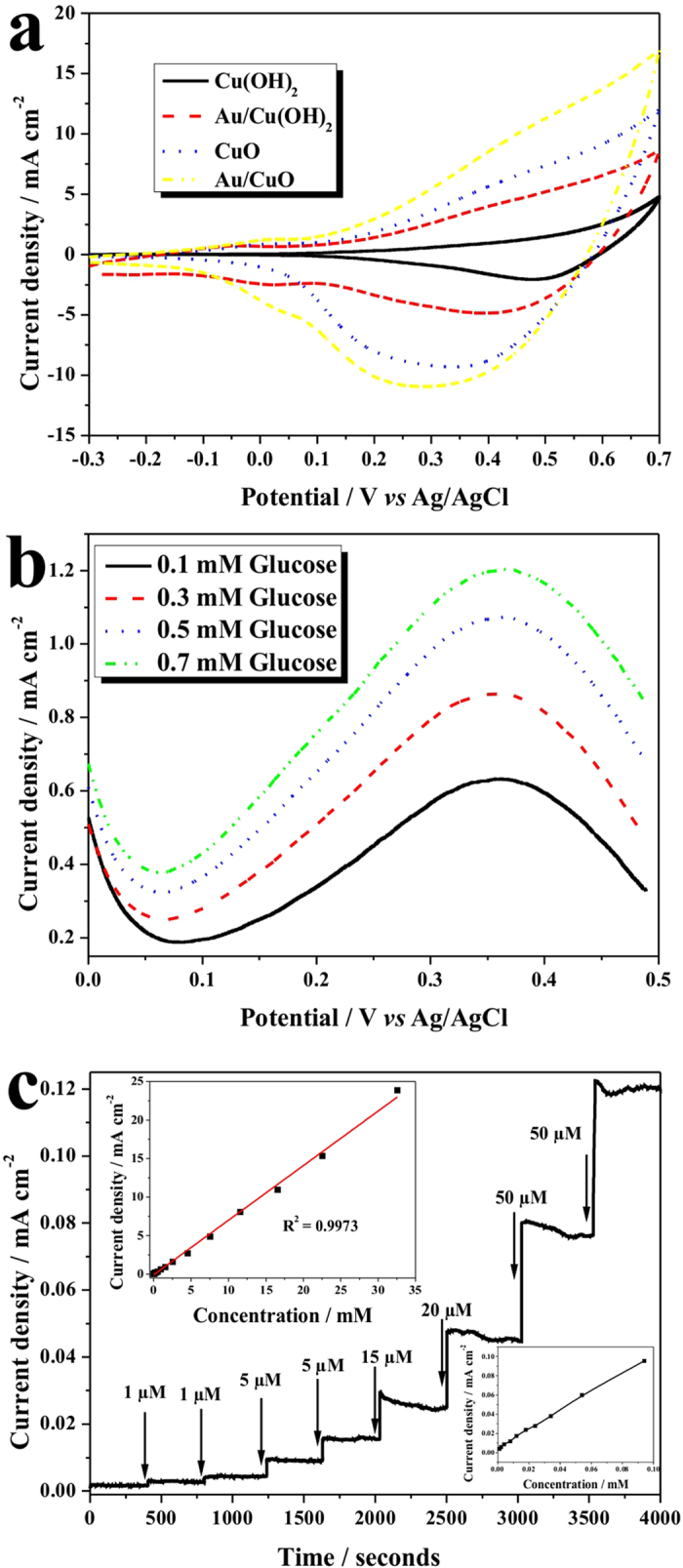
Electrochemical nonenzymatic detection. (**a**) Cyclic voltammograms of the Cu(OH)_2_, Au/Cu(OH)_2_, CuO and Au/CuO in 1.0 M NaOH with scan rate of 50 mV s^-1^; (**b**) linear sweep voltammograms of the Au/CuO electrode in various glucose concentrations with scan rate of 50 mV s^-1^ by subtracting the blank response in 1.0 M NaOH; (**c**) amperometric responses of the Au/CuO electrode with successive addition of glucose at 0.35 V *vs* Ag/AgCl, the top-left inset is current-glucose concentration calibration curve, and the bottom-right inset is the calibration curve in low glucose concentrations.

The electrochemical properties of the Cu(OH)_2_, Au/Cu(OH)_2_, CuO and Au/CuO samples were investigated with a cyclic voltammetry (CV) method in 1.0 M NaOH solution with scan rate of 50 mV s^-1^ and working area of 1.0 cm^2^. As shown in Fig. 5a, all samples presented oxidation peak in range of 0.3 to 0.5 V, which can be attributed to the conversion of Cu(II) to Cu(III) which, once formed, may then act as electron transfer media for the oxidation of glucose^48^,^49^. Among all samples, the Au/CuO displayed the highest anodic current density and lowest oxidation potentials, implying its highest electrocatalytic performance. This result was expected due to the good conductivity of the Au nanostructures and high catalytic activity of the CuO nanowires.

A series of linear-sweep voltammograms (LSV) were recorded on the Au/CuO electrode at various concentrations of glucose and presented in Fig. 5b and in Supplementary Fig. S7, with the blank response in NaOH solution being subtracted. Clearly, with an increase in glucose concentration, the anodic current increased, peaked at 0.35 V *vs* Ag/AgCl before declining thereafter. As a result, the potential of 0.35 V *vs* Ag/AgCl was selected as sensing voltage for subsequent amperometric tests so to optimize the electrocatalytic response as well as obtain the best sensitivity. A typical current-time (*I*-t curve) of the Au/CuO sensor electrode was presented in Fig. 5c, which shows the Au/CuO sensor produced an excellent amperometric response with a short response time, in response to addition of glucose with different concentrations. A calibration curve was further plotted and presented in the top-left inset of Fig. 5c, which exhibited a high sensitivity of 708.7 μA mM^-1^ cm^-2^, and the calibration curve in low concentration was presented in the bottom-right inset of Fig. 5c, both of which helped to demonstrate a low detection limit of 0.3 μM (S/N = 3), and a wide linear range. The *I*-t curves of the Cu(OH)_2_, Au/Cu(OH)_2_, CuO and Au/CuO samples were recorded to determine their amperometric sensing properties (Supplementary Fig. S8-11), and the corresponding sensitivity, linear range and detection limit are summarized in Table 1. As expected, the Au/CuO sensor presented superior performance to others due the rational combination of the noble metal and metal-oxide within a desirable nano-framework.

## Discussion

**Figure 6:**
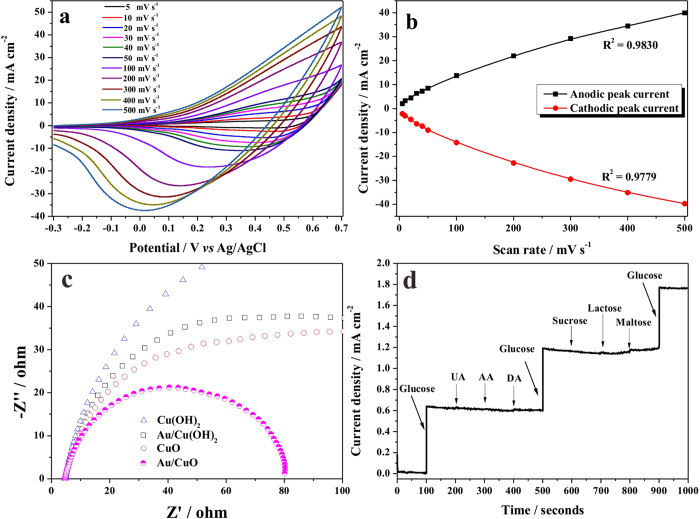
Electrochemical characterization. (**a**) Cyclic voltammograms obtained at Au/CuO nanocauliflower electrode in presence of 1 mM glucose in 1.0 M NaOH at different scan rates, (**b**) the relationship between peak current with scan rate. (**c**) Nyquist plots of Cu(OH)_2_, Au/Cu(OH)_2_, CuO, and Au/CuO samples collected at open circuit potential. (**d**) Anti-interference property of the Au/CuO electrode with initial addition of 1.0 mM glucose and 0.05 mM of UA, AA, and DA, and then again 1.0 mM gluocse, followed by addition of 0.02 mM sucrose, lactose, and maltose, and last addition of 1.0 mM glucose.

The CV responses of the Au/CuO electrode at different scan rate were investigated (Fig. 6a) to confirm the redox reaction model. As presented in Fig. 6b, both anodic and cathodic peak currents varied linearly with potential scan rate in the range of 5 to 500 mV s^-1^, which suggested that the redox reaction is a surface-confined process^50^,^51^, and the glucose molecules were direct oxidized on the surface of composite electrode and the electron were directly transferred, without other mediators.

Electrochemical impedance spectra (EIS) measurement is a powerful tool for studying the electronic properties on the electrode interface. The EIS measurements were carried out to demonstrate the enhancement of conductivity after Au loading, covering the frequency of 10^5^–0.1 Hz interval using an amplitude of 10 mV at the open circuit potential of the system. Fig. 6c presents Nyquist plots for the Cu(OH)_2_, Au/Cu(OH)_2_, CuO, and Au/CuO samples, respectively. Semicircles in Nyquist plots convey information on charge transfer process as the diameters of the semicircles are equal to charge transfer resistance of a sample^52^. As depicted in Fig. 6c, the Au/CuO sample exhibited the smallest semicircular diameter, indeed confirmed the accelerated electron transfer in the presence of the Au nanostructures as the Au/CuO exhibited the lowest resistance among all samples.

Anti-interference property s another essential parameter for nonenzymatic glucose sensor as a good selectivity ensures high accuracy. The selectivity of the Au/CuO sensor was tested with various potentially interfering reagents. As shown in Fig. 6d, the addition of 1.0 mM of glucose resulted in a quick and significant current increase, whereas an addition of 0.1 mM of uric acid (UA), ascorbic acid (AA), dopamine (DA), and 0.05 mM such saccharides as sucrose, lactose, and maltose did not cause observable current changes. Considering that the concentrations of these tested interfering substances in plasma are substantially lower than that of glucose^53^,^54^, the Au/CuO sensor possesses a very favorable selectivity toward glucose detection.

To further evaluate the reproducibility, five Au/CuO electrodes were investigated at 0.35 V to compare their amperometric current response, and a good relative standard deviation of 1.56% was achieved, confirming the good reproducibility. In addition, the long-term stability of the Au/CuO electrodes were also tested by studying the current response intermittently in a period of 30 days, and a good repeatability was found for the same glucose sample, suggesting the Au/CuO electrode is quite stable for the glucose sensing.

In summary, we have successfully synthesized the Au/CuO nanocauliflower electrode for highly sensitive nonenzymatic glucose sensing, and demonstrated that the noble metal/metal oxide composite with binder-free connection present excellent electrocatalytic activity and high sensitivity. This study is the meaningful first step in exploring noble metal/metal oxide composite for nonenzymatic sensing.

## Methods

### Synthesis of Au/CuO nanowire arrays

All the chemicals were of analytical grade and used as purchased without further purification. Copper foil (thickness of 0.1 mm) were supplied by Hebei Jinjia Metal Materials Ltd. Co (P. R. China). HAuCl_4_, sodium hydroxide, glucose, dopamine, ascorbic acid, uric acid, sucrose, lactose, and maltose were supplied by Macklin Inc, Shanghai, China. The Cu foil was anodized in an alkali solution (3 M NaOH) for 30 min under 10 mA cm^-2^ to form Cu(OH)_2_ nanowire. The temperature of the electrochemical cells was maintained at 25 °C for all experiments. The as-anodized nanowire was annealed at 180 ^o^C for 1 h to converted Cu(OH)_2_ to CuO. The nanowire arrays were dipped into HAuCl_4_ solution (pH = 6.0) under simulated solar light irradiation (100 mW cm^-2^) for 30 min for Au deposition.

### Materials Characterization

The morphologies electrodes were characterized by scanning electron microscopy (SEM, FEI, Quanta 600). The crystalline structure of the samples was analyzed by X-ray diffraction (XRD) (Bruker D8 Discover diffractometer, using Cu Kα radiation (1.540598 Å)). The chemical compositions and status were analyzed by X-ray Photoelectron Spectroscopy (XPS) with an Axis Ultra instrument (Kratos Analytical) under ultrahigh vacuum (<10^−8^ torr) and by using a monochromatic Al Kα X-ray source. The adventitious carbon 1 s peak was calibrated at 284.8 eV and used as an internal standard to compensate for any charging effects.

### Electrochemical nonenzymatic detetion

All the electrochemical measurements including cyclic voltammetry, chronoamperometry and electrochemical impedance spectroscope were performed with CHI 660E electrochemical working station in a three-electrode system with nanowire electrodes as working electrode (geometrical area in solution is 1.0 cm^2^), a platinum foil as the counter electrode, and Ag/AgCl with saturated KCl solution as the reference electrode.

## Additional Information

**How to cite this article**: Li, Z. *et al.* Rational design of binder-free noble metal/metal oxide arrays with nanocauliflower structure for wide linear range nonenzymatic glucose detection. *Sci. Rep.* **5**, 10617; doi: 10.1038/srep10617 (2015).

## Supplementary Material

Supporting Information

## Figures and Tables

**Table 1:** Comparison of performance of different nonenzymatic glucose sensors

**Samples**	**Sensitivity /μA mM**^**-1**^ **cm**^**-2**^	**Linear range**	**Detection of Limit / μM**
Cu(OH)_2_	334.7	24 μM-30 mM	8.3
Au/Cu(OH)_2_	390.2	14 μM-30 mM	8.0
CuO	442.0	8.0 μM-30 mM	6.2
Au/CuO	708.7	1.0 μM-30 mM	0.3
