# Supplementary material for: Rational design of binder-free noble metal/metal oxide arrays with nanocauliflower structure for wide linear range nonenzymatic glucose detection
Source: Sci Rep. 2015 Jun 12;5:10617. doi: 10.1038/srep10617 (PMC4464387; doi:10.1038/srep10617)
Supplement: Supporting Information [file srep10617-s1.pdf]

Supplementary Information

Rational design of binder-free noble metal/metal oxide arrays with nanocauliflower structure for wide linear range nonenzymatic glucose detection

Zhenzhen Li,[1] Yanmei Xin,[1] Zhonghai Zhang,[1] Hongjun Wu,[2] & Peng Wang [3]

[1] School of Chemistry and Molecular Engineering, East China Normal University, 500 Dongchuan Road, Shanghai 200241, China.

[2] Provincial Key Laboratory of Oil & Gas Chemical Technology, College of Chemistry & Chemical Engineering, Northeast Petroleum University, Daqing 163318, China.

[3] Water Desalination and Reuse Center, Biological and Environmental Sciences and Engineering Division, King Abdullah University of Science and Technology, Thuwal, Saudi Arabia

Correspondence and requests for materials should be addressed to Z.Z (email: zhzhang@chem.ecnu.edu.cn)

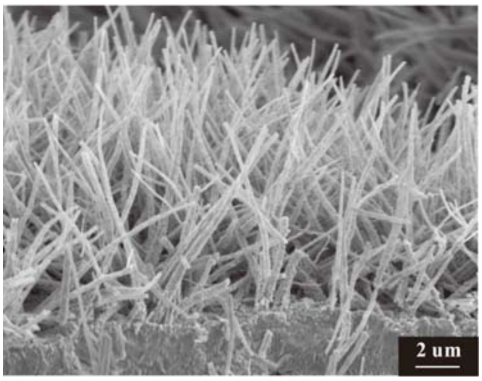

Fig S1. Cross-sectional SEM image of CuO.

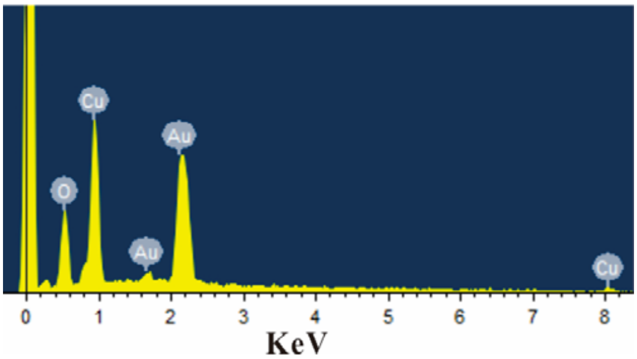

Fig S2. EDS of Au/CuO sample.

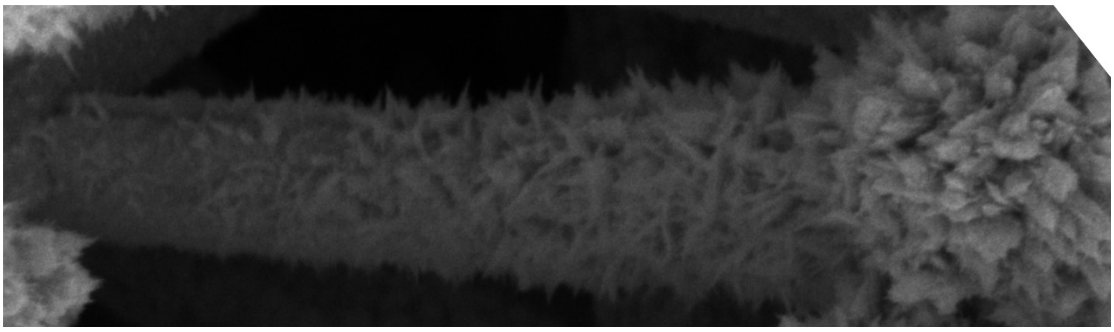

Fig S3. High magnificent SEM image of single Au/CuO nanowire.

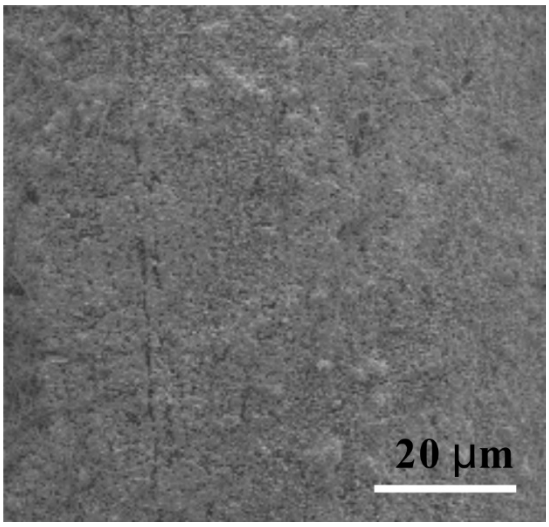

Fig. S4 SEM image of $Cu(OH)_2/Cu$ electrode through photocatalytic reduction action in aqueous $HAuCl_4$ solution with pH value of 3.0. All the $Cu(OH)_2$ nanowires have been removed in this process.

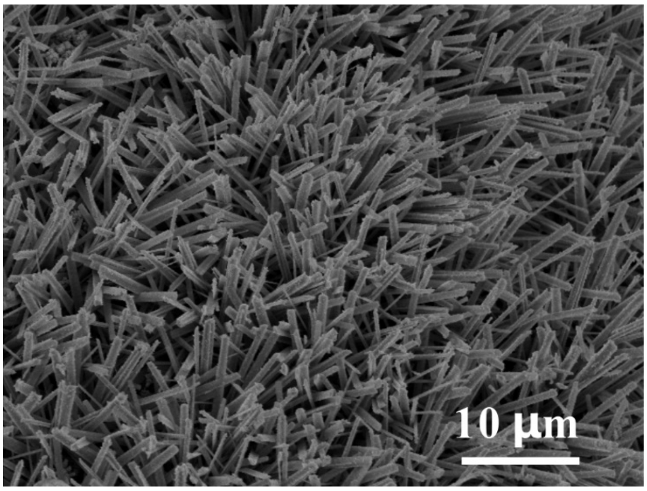

Fig. S5 SEM image of $Cu(OH)_2$/Cu electrode through photocatalytic reduction action in aqueous $HAuCl_4$ solution with pH value of 9.0. No obvious Au nanoparticles on the nanowire surface.

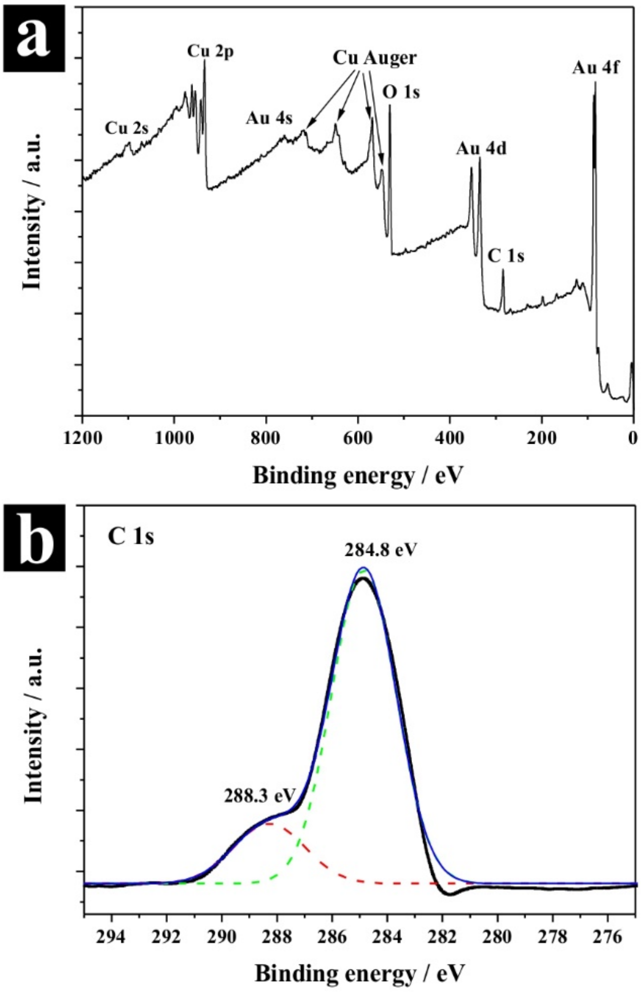

Fig. S6 XPS survey (a) and C 1s core level of Au/CuO sample.

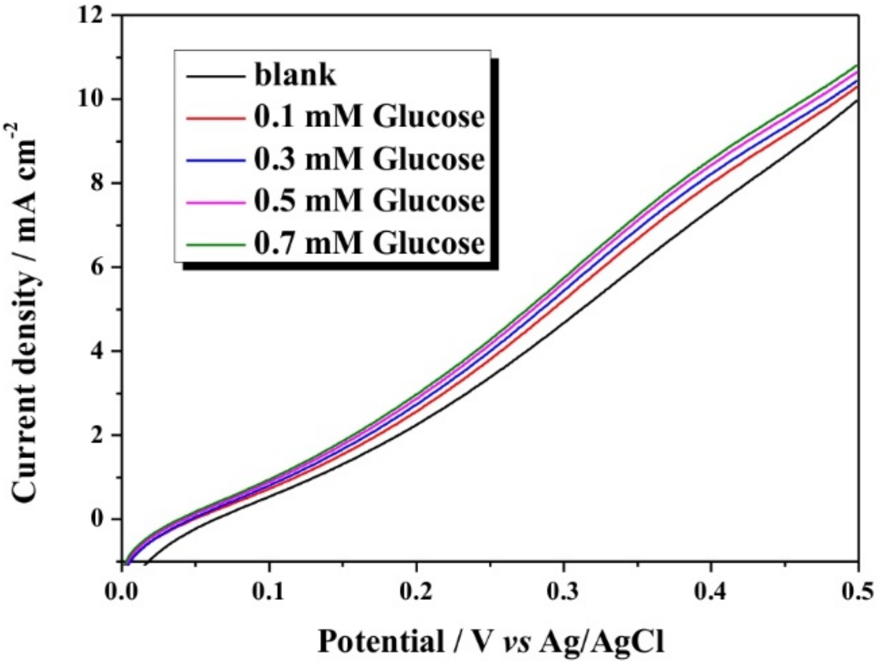

Fig. S7 Linear-sweep voltammograms collected on Au/CuO nanocauliflower electrode with different glucose concentrations in 1.0 M NaOH with scan rate of 50 mV s^{-1}.

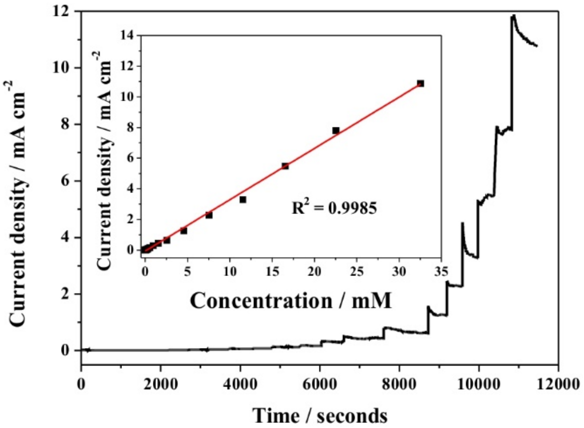

Fig. S8 Amperometric responses of $Cu(OH)_2$ electrode with successive addition of glucose at 0.35 V *vs* Ag/AgCl, the inset is current-glucose concentration calibration curve.

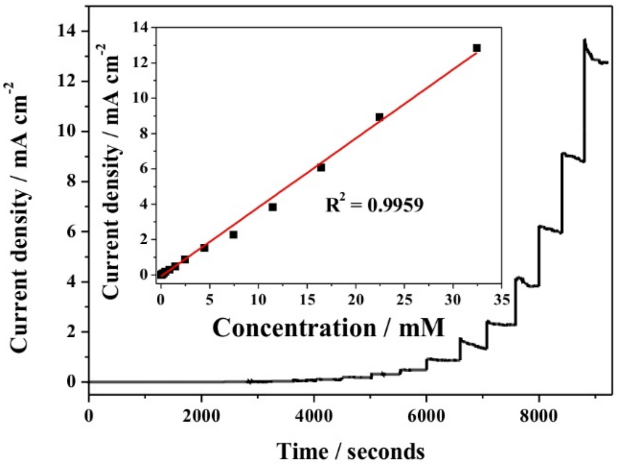

Fig. S9 Amperometric responses of Au/$Cu(OH)_2$ electrode with successive addition of glucose at 0.35 V *vs* Ag/AgCl, the inset is current-glucose concentration calibration curve.

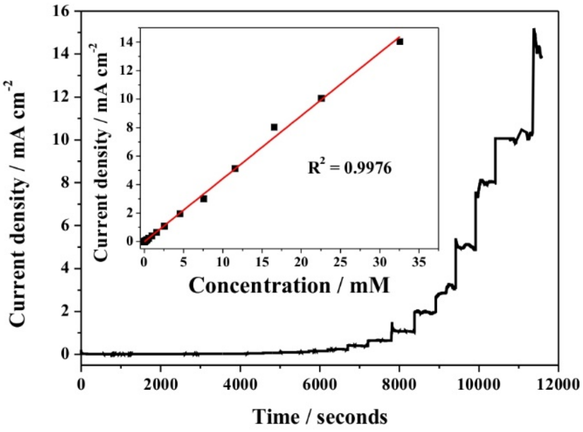

Fig. S10 Amperometric responses of CuO electrode with successive addition of glucose at 0.35 V *vs* Ag/AgCl, the inset is current-glucose concentration calibration curve.

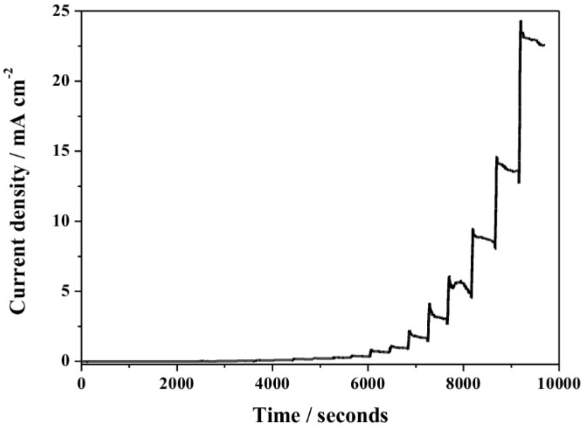

Fig. S11 Amperometric responses of Au/CuO nanocauliflower electrode with successive addition of glucose at 0.35 V *vs* Ag/AgCl in wide concentration range.
